# A Closer Look at the Emotional Intelligence Construct: How Do Emotional Intelligence Facets Relate to Life Satisfaction in Students Involved in Bullying and Cyberbullying?

**DOI:** 10.3390/ejihpe11030051

**Published:** 2021-07-09

**Authors:** Cirenia Quintana-Orts, Sergio Mérida-López, Lourdes Rey, Natalio Extremera

**Affiliations:** 1 Department of Educational and Developmental Psychology, University of Seville, 41018 Seville, Spain; 2 Department of Social Psychology, Social Work, Social Anthropology and East Asian Studies, University of Malaga, 29071 Malaga, Spain; sergioml@uma.es (S.M.-L.); nextremera@uma.es (N.E.); 3 Department of Personality, Evaluation and Psychological Treatment, University of Malaga, 29071 Malaga, Spain; lrey@uma.es

**Keywords:** emotional intelligence, well-being, bullying, cyberbullying, adolescence

## Abstract

Adolescents’ perception of their own emotional abilities has been related to psychological adjustment and well-being. However, there are still few studies focusing on specific emotional dimensions in relation to bullying and well-being in adolescence. This study analysed the relationship between emotional intelligence (EI) facets, satisfaction with life, bullying and cyberbullying in adolescents. The sample consisted of 3520 high school students (51.5% females) aged between 12 and 18 years (M = 14.37; SD = 1.67). The correlation analyses showed that the majority of EI facets were positively related to satisfaction with life and negatively with both types of violence. As was expected, bullying and cyberbullying victims and bully–victims scored lower in satisfaction with life and the majority of EI facets. Controlling for sex, age, and grade, self-emotion appraisal, use of emotions and regulation of emotion were the best predictors of life satisfaction in bully–victims of bullying and cyberbullying. Finally, we discuss the relevance of these findings for clinical and educational practice on EI seeking to promote subjective well-being among adolescents involved in bullying and cyberbullying.

## 1. Introduction

Adolescence is characterised as a period of vulnerability in which demanding and challenging life events increase and well-being may be at risk [1,2]. One of the most stressful situations that negatively affects adolescents’ well-being is bullying [3]. As a distinct form of aggressive behaviour, bullying is conceptualised as ‘(1) intentional negative behaviour that (2) typically occurs with some repetitiveness and is (3) directed against a person who has difficulty defending himself or herself’ [4]. The wide-ranging use of communication technologies and social networks has developed a kind of bullying through cyberspace called cyberbullying [5]. Both aggressive phenomena share common features, such as the extension and repetition in time, the dominance–submission schema between victims and aggressors, the law of silence and several psychological effects appearing among involved adolescents [6]. Despite the similarities, some authors examine both forms as distinct phenomena, with their specific predictors and unique features [7]. 

Involved adolescents can play different roles, such as being a victim (i.e., who suffered victimisation), bully (i.e., who starts and leads the aggression) or being involved with a dual role of bully–victim (i.e., both a victim and an aggressor) in both bullying and cyberbullying. Each role of both forms of aggression has been widely linked to socio-emotional and behavioural problems [8,9,10,11]. Comparing different bullying roles, empirical studies have established that victims and bully–victims are frequently the profiles that are at higher risk of suffering from psychological and emotional sequels [12,13,14]. Regarding well-being, people involved in different kinds of bullying report a decrease in subjective levels of well-being [15,16]. In general, life satisfaction, defined as the cognitive component of well-being [17], has been negatively related to involvement in bullying and cyberbullying [10,15,18]. For example, victims of cyberbullying are found to report lower levels of psychological well-being in comparison to non-involved students [19]. Similarly, victims of bullying have reported lower scores for different well-being indicators [18]. 

One important construct that has been shown to have an impact on improved well-being and reduced aggressive behaviours is emotional intelligence (EI). From the ability model, EI is understood as a set of interrelated skills: ‘(a) perceive emotions accurately, (b) use emotions to accurately facilitate thought, (c) understand emotions and emotional meanings and (d) manage emotions in themselves and others’ [20]. EI theory has demonstrated that individuals who are competently capable of processing information about emotions and use this information as a guide to regulate their feelings report higher emotional adjustment and greater social relationships [21,22]. In short, adolescents with higher EI are able to adaptively cope with distressing experiences and the hassles of everyday life more easily [23,24], reporting higher scores in well-being outcomes [25,26]. 

Thus, prior research suggests that deficits in EI might be key factors involved in situations of school bullying and cyberbullying [27,28] and their associated negative consequences in well-being [7,29,30]. On the one hand, aggressors who bully in both traditional bullying and cyberbullying have been found to report more difficulties in the dimensions of understanding and regulating emotions [31]. However, Segura and colleagues [32] found no deficits in the dimensions of mood attention and emotional clarity among cyberaggressors. On the other hand, pure victims of both phenomena were found to indicate reduced emotional adjustment in comparison to the uninvolved, with lower attention, emotional clarity and regulation of emotions [31]. In general, the previous scientific literature about specific EI components in different bullying roles is still mixed and scarce. 

Regarding life satisfaction, some research has established that EI shows predictive validity on subjective well-being indicators, even when different well-known constructs such as personality, positive and negative affects or self-esteem are taken into consideration [21,25,33,34]. More specifically, emotional clarity and mood repair have been found to show predictive value on life satisfaction [33,34]. In bullying and cyberbullying contexts, recent works suggest that adolescents with higher EI abilities scored higher in life satisfaction than their counterparts with lower EI [30,35]. 

Although there is considerable evidence that adolescents with high EI are more likely to report greater levels of life satisfaction and well-being, even in contexts of bullying and cyberbullying [29,30,36], prior findings have focused mainly on EI as a global construct or have relied on instruments focused specifically on intrapersonal facets (e.g., using the Trait Meta-Mood Scale [31]). In general, most of these existing research findings are focused on one or two specific bullying roles (i.e., pure victims and pure aggressors; or dual roles of traditional–online bullies or victims). To extend the knowledge about the impact of EI facets on life satisfaction in situations of bullying and cyberbullying, the main aim of the present study is to examine the specific EI dimensions (including other-focused appraisal) in different roles (i.e., uninvolved, victims, perpetrators and bully–victims) in a large sample of adolescents. 

Based on the research approaches aforementioned, we hypothesised that:

**Hypothesis** **(H1).** *Higher scores on EI facets will be related to higher satisfaction with life and lower bullying and cyberbullying scores.*

**Hypothesis** **(H2).** *There will be differences in satisfaction with life scores and EI facets, with victims and bully–victims of both bullying and cyberbullying presenting the lowest scores in comparison to the non-involved.*

**Hypothesis** **(H3).** *EI facets will be significant predictors of satisfaction with life in victims and bully–victims of both phenomena.*

## 2. Methods

### 2.1. Participants and Procedure

The study sample comprised 3520 adolescents (51.5% girls) with a mean age of 14 years (M = 14.37, SD = 1.67, range 12–18 years). A convenience sample of adolescents from fifteen high schools located in Southern Spain participated in this cross-sectional study. Most participants were Spanish (88.7%), and other nationalities included English (1.6%), Moroccan (1.3%) and Argentinian (0.8%). The distribution of academic level was as follows: 22.8% were attending classes of the first course of compulsory secondary education; 23.1% were attending classes of the second course of compulsory secondary education; 19.1% were attending the third course; 14.8% were attending the fourth course; the remainder (20.2%) were attending classes in high school (*Bachillerato* in Spanish). 

Data for this study were collected as part of a larger project on positive personal resources against bullying and cyberbullying in Spanish adolescents, after approval of the research ethics committee of the hosting university (62-2016-H). To recruit the sample, a convenience sampling method was adopted by the research team, who contacted several high schools asking them whether they would be willing to take part. Before data collection, consent was received from the centres and parents. The administration of the instruments was carried out by a member of the research group in the presence of a member of the teaching staff and during a regular class period of 50 minutes. Previously, students were informed about the instructions to participate and fill out the pencil-and-paper self-report instruments. Voluntary and anonymous participation was encouraged. 

### 2.2. Instruments

#### 2.2.1. Bullying and Cyberbullying Aggression and Victimisation

The European Bullying Intervention Project Questionnaire (EBIPQ [37]) was used to evaluate traditional bullying aggression and victimisation. This instrument comprises 14 items using a 5-point Likert scale to capture the frequency of bullying over the previous two months, ranging from 0 (no) to 4 (yes, more than once a week). Regarding bullying aggression, this scale comprises seven items on participating in several bullying behaviours (e.g., ‘I have hit, kicked or pushed someone’). With regard to bullying victimisation, seven items evaluate the frequency of having participated in behaviours involving physical and verbal behaviours as well as social exclusion (e.g., ‘someone has hit, kicked or pushed me’). The Spanish version was used [37]. In the present study, Cronbach’s alpha was 0.82 for victimisation and 0.77 for aggression.

#### 2.2.2. Cyberbullying Aggression and Victimisation

The European Cyberbullying Intervention Project Questionnaire (ECIPQ [38]) was used to measure cyberbullying aggression and victimisation. This scale consists of 22 items assessing the frequency of behaviours to assess two dimensions of cyberbullying in the last two months. Each item is measured using a 5-point Likert-type scale ranging from 0 (no) to 4 (yes, more than once a week). On the one hand, the scale evaluates 11 items regarding cyberaggression behaviours (‘I threatened someone with messages on the internet’). On the other hand, the scale assesses 11 items involving cybervictimisation behaviours (e.g., ‘Someone has threatened me through messages on the internet or SMS’). The Spanish version was used [37]. In this study, Cronbach’s alpha was 0.85 for cybervictimisation and 0.82 for cyberaggression. 

#### 2.2.3. Emotional Intelligence

The Wong and Law Emotional Intelligence Scale (WLEIS [39]) was used to assess EI. This scale comprises 16 items organised into four-item subscales following the ability EI model: self-emotion appraisal, other-emotion appraisal, use of emotion and regulation of emotion. Additionally, the scores for the four subscales are summed to provide an overall EI score, with higher scores indicating greater EI. The instrument is made up of items such as ‘I always know what my friends’ emotions are because of their behaviour’ (other-emotion appraisal) and ‘I can easily calm myself down when I feel angry’ (regulation of emotion). Participants are given a 7-point Likert-type scale ranging from 1 (totally disagree) to 7 (totally agree). The Spanish version was used [40]. In this study, Cronbach’s alpha was: 0.75 for self-emotion appraisal, 0.71 for other-emotion appraisal, 0.77 for use of emotion and 0.80 for regulation of emotion. Cronbach’s alpha for overall EI was 0.87.

#### 2.2.4. Satisfaction with Life

The Satisfaction with Life Scale (SWLS [41]) was used. This scale comprises five items requiring participants to state their agreement with self-referenced statements about global life satisfaction. Items are scored with a 7-point Likert-type scale ranging from 1 (strongly disagree) to 7 (strongly agree). One example item is ‘I am satisfied with my life’. The well-validated Spanish version was used [42]. In this study, Cronbach’s alpha was 0.81. 

### 2.3. Analytic Plan 

Data were analysed using SPSS version 22. First, descriptive statistics and Pearson bivariate correlation analyses were conducted to test H1—that is, to examine the associations among traditional bullying, bullying victimisation, cyberaggression, cybervictimisation, overall EI, EI dimensions and satisfaction with life (see Table 1). Second, the criterion proposed by Elipe and colleagues [43] was used to classify participants’ involvement in different roles regarding traditional bullying. These roles were calculated taking into consideration their answers regarding traditional bullying and cyberbullying aggression and victimisation [12,44]. A student was classified as non-involved if they marked the 0 (never) or 1 (yes, once or twice) option in all items regarding bullying, whereas they were classified as a bullying perpetrator if they answered 2 or more (once or twice a month or more) to any item on bullying aggression together with 0 or 1 to all the items on bullying victimisation. Adolescents were classified as victims if they responded using the reverse pattern with the items describing aggression and victimisation behaviours. The same criteria were applied for classifying students in cyberbullying roles. 

Third, the assumptions for using ANOVA were examined to ensure no important violation. Both skewness and kurtosis values of the main variables were found to be within acceptable limits for a normal distribution (less than 3 and 8 points, respectively), whereas the Durbin–Watson contrast values were obtained for the analyses of each group (all values between 1.5 and 1.9), supporting that there was independence. After performing the Levene test for equality of variances, scores in overall EI, EI dimensions and satisfaction with life were compared with one-way ANOVA for different groups regarding involvement in traditional bullying and cyberbullying (i.e., this analytic plan was used for testing H2). Further, post hoc Scheffe pairwise comparisons were performed where variance was homogeneous, whereas post hoc Games–Howell pairwise comparisons were performed where variance was heterogeneous. Moreover, Cohen’s *d* was calculated with 95% confidence intervals to compare groups and to indicate the effect size of differences between groups. 

Fourth, multiple linear regression analyses were conducted to test H3—that is, to examine whether EI dimensions were predictive of satisfaction with life among adolescents involved in problematic roles such as victims and bully–victims [12,44]. Before conducting the multiple linear regression, the assumptions of linear regression were fulfilled for both the victims and bully–victims groups in the current study. Predicted probability plots, residuals scatterplots and variance inflation factor (VIF) < 1.72 and tolerance > 1.01 were used to test, respectively, the normality, homoscedasticity and non-multicollinearity assumption for regression. Age and sex were used as covariates in regression analyses as they have been found to be relevant sociodemographic factors regarding bullying and cyberbullying outcomes [13,14]. According to Cohen’s *f*^2^ for linear regression analyses, effect sizes for the variance explained by the predictors were considered to be small (*f*^2^ = 0.02), medium (*f*^2^ = 0.15) and large (*f*^2^ = 0.35).

## 3. Results

### 3.1. Descriptive Statistics

Table 1 shows descriptive statistics and bivariate correlations among the main study variables. As shown, both bullying and cyberbullying aggression and victimisation were negatively associated with satisfaction with life. Overall EI was negatively related to both bullying and cyberbullying aggression and victimisation, whereas it was positively associated with satisfaction with life. Regarding the associations among EI dimensions and bullying and cyberbullying aggression, significant and negative relationships were found for the majority of EI dimensions. The only exception was the non-significant association between use of emotions and cyberaggression. Finally, the majority of EI dimensions were negatively associated with cybervictimisation. Among the exceptions to this pattern, other-emotion appraisal was not significantly related to cybervictimisation, and other-emotion appraisal was positively related to traditional victimisation. Taken together, the results supported H1. 

### 3.2. Testing Differences among Groups Involved in Traditional Bullying

In order to test H2, scores in EI dimensions and satisfaction with life were tested among different bullying roles involved in traditional bullying and cyberbullying. Regarding differences among groups of involvement in traditional bullying, results are shown in Table 2. 

As shown, results showed significant differences among groups for satisfaction with life and all EI dimensions except for other-emotion appraisal. Scheffe’s post hoc tests revealed that uninvolved adolescents scored significantly higher in overall EI than victims (*d* = 0.19, 95% CI = 0.08–0.29), perpetrators (*d* = 0.21, 95% CI = 0.00–0.39) and bully–victims (*d* = 0.26, 95% CI = 0.12–0.37). Likewise, uninvolved students scored higher in regulation of emotion than victims (*d* = 0.28, 95% CI = 0.23–0.55), perpetrators (*d* = 0.36, 95% CI = 0.20–0.77) and bully–victims (*d* = 0.37, 95% CI = 0.35–0.70).

Games–Howell’s post hoc tests revealed significant differences among groups for self-emotion appraisal and use of emotion. Results showed that uninvolved adolescents scored significantly higher in self-emotion appraisal than victims (*d* = 0.14, 95% CI = 0.04–0.30) and bully–victims (*d* = 0.18, 95% CI = 0.07–0.36). Similarly, uninvolved adolescents showed significantly higher scores in use of emotion than victims (*d* = 0.18, 95% CI = 0.10–0.37) and bully–victims (*d* = 0.18, 95% CI = 0.08–0.39). No significant differences were found regarding other-emotion appraisal. 

With regard to satisfaction with life, Games–Howell’s post hoc tests showed that uninvolved students scored significantly higher than victims (*d* = 0.40, 95% CI = 0.36–0.63), perpetrators (*d* = 0.20, 95% CI = 0.00–0.48) and bully–victims (*d* = 0.51, 95% CI = 0.48–0.78). Finally, perpetrators scored higher in satisfaction with life than victims (*d* = 0.20, 95% CI = 0.00–0.51) and bully–victims (*d* = 0.30, 95% CI = 0.13–0.65).

Taken together, the results supported H2, with victims and bully–victims of bullying reporting the lowest scores in satisfaction with life and each EI facet, except for other-emotion appraisal, when compared with uninvolved adolescents. 

### 3.3. Testing Differences among Groups Involved in Cyberbullying

To test H2, scores in EI dimensions and satisfaction with life were examined among different bullying roles involved in traditional cyberbullying. Results are shown in Table 3. 

As Table 3 shows, significant differences among groups for satisfaction with life and all EI dimensions were found. On the one hand, Scheffe’s post hoc tests revealed that uninvolved adolescents scored significantly higher in overall EI than cybervictims (*d* = 0.22, 95% CI = 0.06–0.37) and cyberbully–victims (*d* = 0.22, 95% CI = 0.02–0.40). Moreover, cybervictims showed higher scores in other-emotion appraisal than cyberaggressors (*d* = 0.28, 95% CI = 0.03–0.60) and cyberbully–victims (*d* = 0.25, 95% CI = 0.09–0.64). Regarding satisfaction with life, uninvolved adolescents scored significantly higher in this variable than cybervictims (*d* = 0.59, 95% CI = 0.55–0.96), cyberaggressors (*d* = 0.25, 95% CI = 0.05–0.59) and cyberbully–victims (*d* = 0.24, 95% CI = 0.05–0.55). Moreover, regarding scores in satisfaction with life, cyberaggressors scored higher than cybervictims (*d* = 0.33, 95% CI = 0.11–0.76) and cyberbully–victims scored higher than cybervictims (*d* = 0.35, 95% CI = 0.14–0.77).

Games–Howell’s post hoc tests revealed significant differences among groups for self-emotion appraisal, regulation of emotion and use of emotion. With regard to self-emotion appraisal, uninvolved adolescents scored significantly higher in this variable than cybervictims (*d* = 0.28, 95% CI = 0.14–0.54) and cyberbully–victims (*d* = 0.25, 95% CI = 0.07–0.56). Similarly, uninvolved students scored higher in regulation of emotion than cybervictims (*d* = 0.28, 95% CI = 0.22–0.67) and cyberbully–victims (*d* = 0.25, 95% CI = 0.11–0.62). Finally, it was found that uninvolved adolescents scored significantly higher in use of emotion than cybervictims (*d* = 0.17, 95% CI = 0.02–0.46). 

### 3.4. Multiple Linear Regression Analyses for Predicting Scores in Satisfaction with Life among Victims and Bully–Victims of Traditional Bullying

To examine H3 and to examine and evaluate the separate contribution of sociodemographic variables (i.e., age and sex), self-emotion appraisal, other-emotion appraisal, use of emotion and regulation of emotion for the prediction of satisfaction with life among the groups involved in traditional bullying and cyberbullying with the lowest scores in EI dimensions and in satisfaction with life (i.e., victims and bully–victims), a set of multiple hierarchical regression analyses was conducted. For the first step, age (continuous variable) and sex (dichotomous variable being coded 1 as male and 2 as female) were entered as covariates. Second, EI dimensions were entered into the regression. Results are separated for traditional bullying and cyberbullying. 

#### 3.4.1. Results for Traditional Bullying and Cyberbullying Victims

Table 4 shows the main results for the hierarchical regression analyses for the groups of victims involved in traditional bullying and cyberbullying. As shown, several EI dimensions were predictive of satisfaction with life after controlling for age and sex, explaining a large (*f*^2^ = 0.45) amount of variance in satisfaction with life. In particular, self-emotion appraisal (B = 0.20, *p* < 0.001), use of emotion (B = 0.38, *p* < 0.001) and regulation of emotion (B = 0.11, *p* < 0.05) reached statistical significance in predicting higher satisfaction with life among victims of traditional bullying. The total model accounted for a large (*f*^2^ = 0.47) amount of variance in satisfaction with life. With regard to cybervictims, a similar pattern of results was found, with the only exception being other-emotion appraisal. In particular, self-emotion appraisal (B = 0.15, *p* < 0.05), use of emotion (B = 0.36, *p* < 0.001) and regulation of emotion (B = 0.13, *p* < 0.05) explained higher satisfaction with life among cybervictims, whereas other-emotion appraisal was a significant negative predictor (B = −0.13, *p* < 0.05). The inclusion of EI facets in the prediction model accounted for a large (*f*^2^ = 0.44) amount of variance in satisfaction with life, with the total model explaining a large (*f*^2^ = 0.45) amount of variance in the dependent variable. In sum, the results partially supported H3 regarding the predictive effects of EI dimensions on satisfaction with life among victims of traditional bullying and cyberbullying. 

#### 3.4.2. Results for Bully–Victims Involved in Traditional Bullying and Cyberbullying 

Table 5 displays the main results for the hierarchical regression analyses for the groups of bully–victims regarding traditional bullying and cyberbullying. Overall, the results showed several EI dimensions to be predictive of satisfaction with life. These dimensions explained a medium (*f*^2^ = 0.24) amount of variance in satisfaction with life. As Table 5 shows, self-emotion appraisal (B = 0.16, *p* < 0.01) and use of emotion (B = 0.31, *p* < 0.001) significantly explained higher satisfaction with life among bully–victims involved in traditional bullying. The total prediction model accounted for a medium (*f*^2^ = 0.25) amount of variance in satisfaction with life. With regard to cyberbully–victims, similar results were found, indicating that self-emotion appraisal (B = 0.15, *p* < 0.05) and use of emotion (B = 0.36, *p* < 0.001) were positive predictors of satisfaction with life. The inclusion of EI facets led to explaining a medium (*f*^2^ = 0.29) amount of variance in satisfaction with life, whereas the total model accounted for a medium (*f*^2^ = 0.31) amount of variance in the outcome. Overall, the results supported H3, showing that two EI dimensions were predictive of satisfaction with life among bully–victims of traditional and cyber-bullying. 

## 4. Discussion and Conclusions

While prior findings have found that EI is typically related to reduced bullying and cyberbullying involvement as well as with increased subjective well-being [25,28], there is a lack of knowledge about the particular role of EI dimensions in these relationships considering different bullying and cyberbullying roles. Thus, this research examined the relationship among EI facets, satisfaction with life, bullying and cyberbullying aggression and victimisation in a sample of adolescents who were classified into different roles according to their degree of participation in both phenomena.

### 4.1. The Relationships among EI Facets, Satisfaction with Life, Bullying and Cyberbullying 

Regarding H1, our results showed that satisfaction with life was negatively related to traditional and online victimisation and aggression, in line with recent research [10,18]. Additionally, global EI and the independent EI facets were positively correlated with life satisfaction, which is in line with prior studies [30,45,46]. Additionally, consistent with previous studies, the majority of EI facets (except other-emotion appraisal) were negatively related to bullying and cyberbullying aggression and victimisation [7,30,47]. In accordance with Schoeps and colleagues [30], our results did not show a significant relation between other-emotion appraisal and cybervictimisation. One possible explanation may be related to the features of cyberbullying, where the emotions are perceived by writing resources such as certain characters and emoticons. Thus, emotional expression, such as the tone of voice and the physical clues (e.g., facial or body expression, etc.), is absent in online communication [48].

### 4.2. Differences in EI Facets among Groups Involved in Bullying and Cyberbullying

With regard to H2, differences in satisfaction with life scores and EI facets were found between roles involved and uninvolved in bullying and cyberbullying. The groups of adolescents involved in bullying and cyberbullying reported significantly lower scores in life satisfaction than the group of uninvolved. Consistent with previous research, traditional and online victims showed the lowest scores in life satisfaction [15,36]. In a traditional setting, similar to pure victims, traditional bully–victims reported the lowest scores of life satisfaction. Moreover, cyberbully–victims scored lower in life satisfaction compared to uninvolved adolescents. These results support former research that has indicated that victims and bully–victims are at increased risk of mental and emotional problems [14]. Considering perpetration, bullying and cyberbullying aggressors reported lower scores on life satisfaction compared to uninvolved adolescents. Similarly, these findings are in accordance with previous research indicating that those adolescents engaging in traditional or cyberbullying aggressive behaviours usually report lower subjective well-being and satisfaction with life [15,31]. 

Regarding the results on the specific EI facets, victims and bully–victims of both phenomena reported deficits in self-emotion appraisal and regulation of emotion in comparison to the non-involved and aggressors. In traditional bullying, victims and bully–victims also indicated deficits in the use of emotion. These findings are in line with previous studies reporting low scores in EI facets among victims of traditional and online bullying in comparison with the uninvolved group [31,36,47]. Our results have not only replicated these prior findings but have also extended prior research on the relationships among EI facets and life satisfaction to the bully–victim group. As mentioned, bully–victims and pure victims generally presented lower emotional and psychological adjustment in comparison with the uninvolved students [13,14]. It is plausible that the bully–victims group indicates similar EI deficits to pure victims groups due to the levels of victimisation, specifically in terms of the reduced ability to understand and manage their own emotions [12,29,32]. 

Unexpectedly, cybervictims were found to report higher levels of other-emotion appraisal compared to bully–victims and aggressors. These results are in line with Segura and colleagues [32], who found that cybervictims presented high emotional attention to their own emotions. However, results show that cybervictims present a higher perception of others’ emotions than other groups involved in cyberbullying. Thus, it is possible that the degree of emotional confusion and the difficulties that cybervictims experience is associated with higher attention to others’ emotions because when they feel emotionally confused, by focusing on others’ emotional information, they may strive to understand what is happening [36]. Therefore, they may choose with whom they share their emotions in an attempt to search for social support online [49,50]. However, considering the lack of correlation between cybervictimisation and other-emotion appraisal, this line of research warrants further investigation.

Regarding perpetrators, our findings showed that traditional aggressors seem to report greater difficulty in the ability to appropriately regulate their emotions compared to the uninvolved. These results are consistent with past studies [36,51] indicating that perpetrators may perceive and use emotions to the same extent as an uninvolved adolescent in a bullying situation, whereas their main emotional abilities deficit may be linked to their ability to regulate emotions. For instance, deficits in managing anger and frustration may make them more likely to express aggressive behaviours [31,36]. However, contrary to the recent study by Segura and colleagues [32], these deficits in emotion regulation were not found for cyberbullying aggressors in comparison to the other roles. By contrast, our results are in line with those of Estévez and colleagues [36], who found that cyberaggressors did not show lower scores in emotion regulation. One plausible explanation for these mixed results could be related to the features underlying cyberbullying (e.g., anonymity and the distance from the victim), which enables moral mechanisms that may facilitate disengaging from the emotional consequences of the aggressive behaviours and, in turn, would make the aggressors less likely to perceive a need to regulate their negative emotions [36,52]. 

### 4.3. The Role of EI Facets for Predicting Satisfaction with Life among Groups at High Risk: Victims and Bully–Victims

Regarding H3, our findings indicated some EI facets as the most significant predictors of life satisfaction among bully–victims and victims, after controlling for sociodemographic variables. For victims and bully–victims of both types of violence, self-emotion appraisal and use of emotion were significant positive predictors of satisfaction with life, which is consistent with previous studies using adult samples [53,54]. The former EI facet relates to the ability to understand and express ones’ own emotions, whereas the latter refers to the ability to make use of one’s own emotions by addressing them toward constructive activities and personal performance [53]. Thus, these two abilities, which enable sense and acknowledge emotional states and moods (self-emotion appraisal) and direct emotional information in more positive and productive directions (use of emotion), may help victims and bully–victims to appreciate more adaptive coping strategies [24]. This may extend their positive experiences and reduce the negative ones, which, in turn, would lead to greater life satisfaction [25,54,55]. 

Our findings indicate that the regulation of emotion was a relevant dimension for predicting satisfaction with life among traditional victims and cybervictims but not for bully–victims of both phenomena. In this sense, the victims’ capacity to regulate their feelings is key in order to enable a more rapid recovery from psychological distress and, in turn, enhance psychological well-being [53,56]. In sum, self-emotion appraisal, use of emotion and regulation of emotion are relevant EI facets that may allow victims of bullying and cyberbullying to use richer resources and adaptive coping strategies, interrupting negative emotions and extending the positive ones to maintain better mental health and higher life satisfaction [23,24,25,34]. 

Interestingly, for cybervictims, lower other-emotion appraisal is related to better satisfaction with life. It is possible that greater emotional attention to another’s emotions in cyberspace implies that cybervictims present more difficulties in properly perceiving and understanding the emotions of the online interactions with others, and this, in turn, may make them more sensitive to the emotions of others, thus affecting their satisfaction with life. It is also possible that other-emotion appraisal could have a negative and unbalanced influence on life satisfaction in the cyberbullying context by prolonging negative moods [57] and affecting symptoms of depression [32]. However, due to the lack of correlation between cybervictimisation and other-emotion appraisal, future studies should explore potential links between these variables. 

Finally, although the effect sizes of the multiple regression analyses predicting levels of life satisfaction are in line with previous findings with general adolescent samples (e.g., [21]), the current study extends the existing knowledge by distinguishing victims’ and bully–victims’ roles in bullying and cyberbullying. Taken together, considering the effect sizes across roles, it seemed that victims of bullying and cyberbullying may benefit most from some of their EI facets (i.e., self-emotion appraisal and use of emotion) to preserve their levels of satisfaction with life in comparison with the adolescents involved in bully–victim role behaviours. Nonetheless, this potential differential pattern should be examined in more depth.

### 4.4. Limitations and Future Lines

There are some limitations that should be noted. Although the use of self-administered questionnaires is a common and inexpensive method to reach larger samples, there are potential biases in the responses, such as the social desirability. Due to the features of this sample, to carry out the generalisation of the results, it is necessary to replicate and extend our analyses with other population samples from different geographical areas. In addition, the causality of the relationships is limited, given the cross-sectional nature of the study. Therefore, it is recommended that future investigations analyse these variables, including longitudinal designs and other measurement instruments, such as peer assessments or evaluations by educators. In this sense, although attention was paid to three important groups involved in bullying and cyberbullying, future studies should examine the co-occurrence of these roles in both phenomena. In the same line, future studies should test the predictive validity of particular EI facets, comparing effect sizes across other involved and non-involved groups (i.e., non-involved adolescents, bystanders, aggressors, etc.), as well as to compare potential sex differences in the results. Finally, it is important to keep in mind that our findings are limited to self-report techniques and quantitative approach analysis. The role of emotions in violent or victim behaviour that are not significant in this study, using other types of interview or observer-rating techniques and qualitative analyses, remains to be determined in further research.

### 4.5. Practical Implications 

Despite the above limitations, our results are coherent with previous literature pointing to the relevance of EI for well-being in applied contexts [25,29,30,33,34]. Previous findings have primarily focused on global EI or have relied on intrapersonal facets using one or two specific bullying roles or focusing on non-involved adolescents. Nonetheless, this research extends the understanding of the relationship between EI, including interpersonal and intrapersonal facets, and life satisfaction by considering relevant bullying and cyberbullying roles. Our results will help school practitioners to gain a more granular understanding of which particular EI dimensions facilitate life satisfaction among students involved in bullying and cyberbullying. Indeed, the results of this study have shown the predictive nature of EI facets in those more vulnerable roles attending to the highest negative impact on life satisfaction—that is, among victims and bully–victims in both contexts. As such, this research presents some practical implications on the prevention and treatment of traditional bullying and cyberbullying, taking into consideration differences in certain EI facets and levels of life satisfaction in specific roles of both phenomena. 

Taken together, our results suggest, on the one hand, the early detection and screening of deficits in the emotional skills of adolescents, allowing the identification of vulnerable profiles that may be more likely to present psychological problems affecting their satisfaction with life. This may help counsellors and clinical psychologists to implement more timely preventive strategies to protect adolescents from the potential detrimental effects of bullying and cyberbullying on life satisfaction. On the other hand, our findings encourage intervention programmes that include the development of specific emotional facets, such as those related to the expression, understanding, use and regulation of emotions. On the basis of the need to promote programmes supporting the socio-emotional development of adolescents [22], education or training in emotional skills should be integrated into bullying and cyberbullying education in schools. Considering our results about the deficits in EI facets and life satisfaction scores from a preventative approach, training emotional skills will help adolescents to manage stressful life events and negative emotions more appropriately, developing more adaptive strategies to extend positive experiences [24,25,55] and, ultimately, to prevent them from getting involved in bullying and cyberbullying [36,58]. Promoting certain EI facets in adolescents will not only enable them to better understand their own emotions, but also provide them with the agency and autonomy required for well-being and resilience [59]. Particularly, it seems that self-emotion appraisal and the use of emotions are important for bullying and cyberbullying victims and bully–victims, whereas regulation of emotions emerged as a relevant facet explaining life satisfaction among bullying victims and bully–victims. Examining these significant facets of EI, particularly the profile of traditional bullying and cyberbullying, will also help school counsellors to improve their tracking of student’s outcomes, tailor interventions to specific emotional needs in the classroom and understand why specific victims or perpetrators may not be responding to preventive or intervention programmes. Therefore, when bullying or cyberbullying situations occur, programmes and treatment on specific EI skills may help adolescents to understand and express their emotions, direct their emotions in positive and productive directions and present better control of their emotions, recovering faster from psychological distress [53]. By training these emotional skills in prevention programmes, adolescents at risk for bullying and/or cyberbullying can not only improve their levels of satisfaction in specific life domains, such as health, relationships and academic performance [22,60], but also successfully develop and maintain a higher sense of satisfaction with their lives.

## Figures and Tables

**Table 1:** Descriptive statistics (means and standard deviations) and bivariate correlations among main variables.

Variable	1	2	3	4	5	6	7	8	9	10
1. Traditional bullying	-	0.460 **	0.574 **	0.389 **	−0.133 **	−0.092 **	−0.057 **	−0.059 **	−0.048 **	−0.110 **
2. Traditional victimisation		-	0.316 **	0.582 **	−0.261 **	−0.117 **	−0.107 **	0.042 *	−0.101 **	−0.167 **
3. Cyberbullying			-	0.602 **	−0.089 **	−0.087 **	−0.084 **	−0.072 **	−0.032	−0.079 **
4. Cybervictimisation				-	−0.183 **	−0.085 **	−0.104 **	0.017	−0.049 **	−0.111 **
5. SWLS					-	0.486 **	0.397 **	0.185 **	0.479 **	0.391 **
6. Overall emotional intelligence						-	0.812 **	0.625 **	0.790 **	0.803 **
7. Self-emotion appraisal							-	0.376 **	0.502 **	0.594 **
8. Other-emotion appraisal								-	0.357 **	0.262 **
9. Use of emotions									-	0.511 **
10. Regulation of emotion										-
Mean	0.37	0.70	0.11	0.18	4.78	4.82	4.99	5.17	4.76	4.36
Standard Deviation	0.41	0.70	0.25	0.36	1.29	0.96	1.20	1.11	1.31	1.40

Note: SLWS = Satisfaction with Life Scale. *N* = 3520. * *p* < 0.05; ** *p* < 0.01.

**Table 2:** Descriptive statistics and differences among the study variables regarding roles of involvement in traditional bullying.

Variables	Groups Involved					
	(1)	(2)	(3)	(4)	F	Significant post hoc comparison
	Uninvolved in bullying	Victims	Perpetrators	Bully–victims		
	(n = 1677)	(n = 961)	(n = 211)	(n = 671)		
	M (SD)	M (SD)	M(SD)	M(SD)		
Overall EI	4.93 (0.94)	4.75 (0.97)	4.73 (0.94)	4.68 (0.96)	14.55 ***	1 > 2,3,4
SEA	5.08 (1.14)	4.91 (1.26)	5.05 (1.19)	4.86 (1.26)	7.23 ***	1 > 2,4
OEA	5.16 (1.08)	5.22 (1.13)	5.10 (1.17)	5.15 (1.12)	1.021	-
UOE	4.88 (1.25)	4.65 (1.35)	4.67 (1.40)	4.65 (1.35)	9.166 ***	1 > 2,4
ROE	4.59 (1.35)	4.20 (1.40)	4.11 (1.35)	4.07 (1.43)	32.557 ***	1 > 2,3,4
SWLS	5.05 (1.18)	4.55 (1.34)	4.81 (1.27)	4.42 (1.31)	54.694 ***	1 > 3; 1,3 > 2,4

Note: *N* = 3520, EI = emotional intelligence, SEA = self-emotion appraisal, OEA = other-emotion appraisal, UOE = use of emotion, ROE = regulation of emotion, SWLS = Satisfaction with Life Scale. *** *p* < 0.001.

**Table 3:** Descriptive statistics and differences among the study variables regarding roles of involvement in cyberbullying.

Variables	Groups Involved					
	(1) Uninvolved in cyberbullying (n = 2797) M (SD)	(2) Cybervictims (n = 327) M (SD)	(3) Cyberaggressors (n = 182) M(SD)	(4) Cyberbully–victims (n = 214) M(SD)	F	Significant post hoc comparison
Overall EI	4.86 (0.95)	4.64 (1.03)	4.72 (0.94)	4.65 (0.96)	8.826 ***	1 > 2,4
SEA	5.05 (1.17)	4.70 (1.34)	4.97 (1.21)	4.73 (1.35)	11.624 ***	1 > 2,4
OEA	5.18 (1.09)	5.34 (1.12)	5.02 (1.19)	4.98 (1.74)	5.916 ***	2 > 3,4
UOE	4.79 (1.29)	4.55 (1.45)	4.72 (1.35)	4.81 (1.34)	3.420 *	1 > 2
ROE	4.43 (1.37)	3.99 (1.52)	4.18 (1.42)	4.07 (1.40)	14.418 ***	1 > 2,4
SWLS	4.89 (1.25)	4.13 (1.34)	4.57 (1.32)	4.59 (1.25)	38.684 ***	1 > 2,3,4; 3,4 > 2

Note: *N* = 3520, EI = emotional intelligence, SEA = self-emotion appraisal, OEA = other-emotion appraisal, UOE = use of emotion, ROE = regulation of emotion, SWLS = Satisfaction with Life Scale. * *p* < 0.05; *** *p* < 0.001.

**Table 4:** Results of multiple linear regression for predicting satisfaction with life among victims of traditional bullying and cyberbullying.

Victims (n = 961)				
**Predictor**	**B**	**SE**	***t***	**BCa 95% CI**
Age	0.00	0.03	0.06	[−0.05, 0.05]
Sex	0.03	0.07	0.39	[−0.12, 0.18]
Self-emotion appraisal	0.20	0.04	5.50 ***	[0.13, 0.28]
Other-emotion appraisal	−0.05	0.04	−1.33	[−0.11, 0.02]
Use of emotion	0.38	0.03	11.73 ***	[0.32, 0.44]
Regulation of emotion	0.11	0.03	3.40 **	[0.05, 0.18]
R^2^ (ΔR^2^)	0.32 (0.31)			
*F* (df)	74.36 *** (4, 954)			
**Cybervictims (n = 327)**				
**Predictor**	**B**	**SE**	***t***	**BCa 95% CI**
Age	−0.03	0.04	-0.67	[−0.11, 0.06]
Sex	0.14	0.13	1.13	[−0.11, 0.39]
Self-emotion appraisal	0.15	0.06	2.39 *	[0.03, 0.27]
Other-emotion appraisal	−0.13	0.06	−2.11 *	[−0.25, −0.01]
Use of emotion	0.36	0.05	6.70 ***	[0.26, 0.47]
Regulation of emotion	0.13	0.05	2.47 *	[0.03, 0.24]
R^2^ (ΔR^2^)	0.31 (0.31)			
*F* (df)	23.83 *** (4, 320)			

Note: SE: Standard error. * *p* < 0.05, ** *p* < 0.01, *** *p* < 0.001.

**Table 5:** Results of multiple linear regression for predicting satisfaction with life among bully–victims of traditional bullying and cyberbullying.

Bully–Victims (n = 671)				
**Predictor**	**B**	**SE**	***t***	**BCa 95% CI**
Age	−0.04	0.03	−1.30	[−0.10, 0.02]
Sex	−0.05	0.09	−0.51	[−0.23, 0.13]
Self-emotion appraisal	0.16	0.05	3.38 **	[0.07, 0.25]
Other-emotion appraisal	−0.08	0.05	−1.82	[−0.17, 0.01]
Use of emotion	0.31	0.04	7.43 ***	[0.23, 0.40]
Regulation of emotion	0.07	0.04	1.75	[−0.01, 0.15]
R^2^ (ΔR^2^)	0.20 (0.19)			
*F* (df)	27.41 *** (4, 664)			
**Cyberbully–Victims (n = 214)**				
**Predictor**	**B**	**SE**	***t***	**BCa 95% CI**
Age	0.02	0.05	0.40	[−0.07, 0.11]
Sex	−0.12	0.16	−0.79	[−0.43, 0.19]
Self-emotion appraisal	0.17	0.07	2.36 *	[0.03, 0.32]
Other-emotion appraisal	0.09	0.07	1.32	[−0.05, 0.23]
Use of emotion	0.22	0.07	3.25 **	[0.09, 0.36]
Regulation of emotion	0.10	0.06	1.66	[−0.02, 0.23]
R^2^ (ΔR^2^)	0.24 (0.23)			
*F* (df)	10.75 *** (4, 207)			

Note: SE: Standard error. * *p* < 0.05, ** *p* < 0.01, *** *p* < 0.001.
